# A rapid benchtop method to assess biofilm on marine fouling control coatings

**DOI:** 10.1080/08927014.2021.1929937

**Published:** 2021-06-21

**Authors:** Simon P. J. Dennington, Alexandra Jackson, Alistair A. Finnie, Julian A. Wharton, Jennifer E. Longyear, Paul Stoodley

**Affiliations:** a National Centre for Advanced Tribology at Southampton (nCATS), Department of Mechanical Engineering, University of Southampton, Southampton, UK; b Marine, Protective and Yacht Coatings, International Paint Ltd., AkzoNobel, Felling, Gateshead, Tyne & Wear, UK; c National Biofilm Innovation Centre (NBIC), University of Southampton, Southampton, UK; d Departments of Microbial Infection and Immunity and Orthopedics Center for Microbial Interface Biology, The Ohio State University, Columbus, Ohio, USA

**Keywords:** Biofilm, coatings, disc, rheometer, sandpaper, roughness

## Abstract

A rapid benchtop method to measure the torque associated with minidiscs rotating in water using a sensitive analytical rheometer has been used to monitor the drag caused by marine fouling on coated discs. The method was calibrated using sandpaper surfaces of known roughness. Minidiscs coated with commercial fouling control coatings, plus an inactive control, were exposed in an estuarine harbour. After 176 days the drag on the fouling control-coated discs, expressed as a moment coefficient, was between 73% and 90% less than the drag on the control coating. The method has potential use as a screen for novel antifouling and drag reducing coatings and surfaces. Roughness functions derived using Granville’s indirect similarity law are similar to patterns found in the general hydrodynamics literature, and so rotational minidisc results can be considered with reference to other fouling drag datasets.

Supplemental data for this article is available online at https://doi.org/10.1080/08927014.2021.1929937 .

## Graphical Abstract

**Figure:**
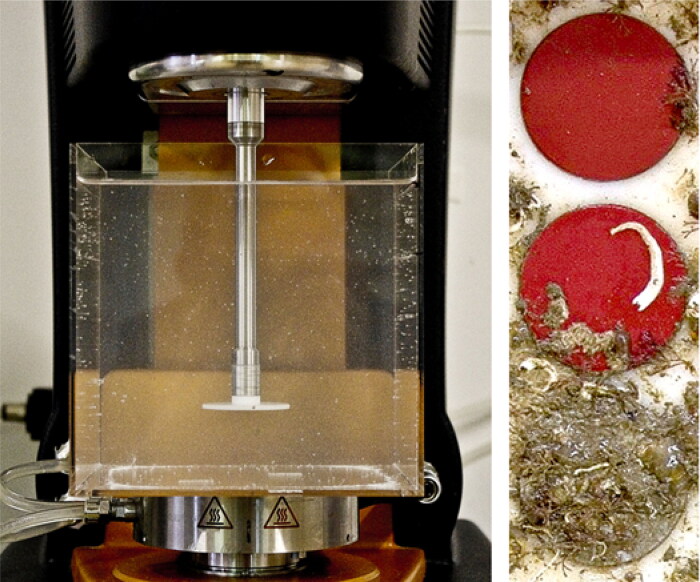

## Introduction

Fouling of ships’ hulls by the attachment of sessile marine organisms greatly increases frictional resistance, reducing the vessel’s speed or increasing fuel consumption if the same speed is maintained (Schultz 2007). Traditional antifouling (AF) coatings for ships contain biocides such as cuprous oxide pigment (Yebra et al. 2004; Finnie and Williams 2010) while foul release coatings (FRCs) are based on elastomeric polymers which have low energy (“non-stick”) surfaces which are biocide-free and can control fouling through their physical properties alone (Galli and Martinelli 2017). Although it remains a challenge for any coating to completely prevent fouling on the hulls of all vessel types and all ship operational profiles, modern fouling control (FC) coatings can provide an effective solution to the problem of fouling by larger organisms (Salta et al. 2013) and marine biofilms (commonly referred to as “slime”) (Muthukrishnan et al. 2014; Benschop et al. 2018) although build-up of diatom slimes are a substantial drawback for predominantly hydrophobic FRCs (Lejars et al. 2012).

Marine biofilms are composed largely of diatoms and bacteria which cement themselves to the surface by means of a sticky extracellular matrix composed of polysaccharides, proteins and other biological macromolecules (Hoagland et al. 1993). These biofilms form thin surface coverings that extend at most a few millimetres above the hull surface and hence are not subject to the same high moment from drag forces that larger growths such as barnacles and trailing weed are, and consequently are less easily removed from FRCs even when the ship is travelling at high speeds (Dobretsov et al. 2013; Townsin and Anderson 2009). Since biofilms can cover large areas of the hull the combined drag penalty on the whole ship may be substantial.

In-service operational data and accurate evaluation of hull fouling conditions are difficult to obtain for ships operating under normal commercial constraints, therefore indirect methods must be used to estimate the frictional drag due to biofilms (Schultz and Myers 2003). Velocity similarity laws for turbulent shear flows, as expounded by Granville (1978, 1982), are then applied to these data to derive roughness functions which may be used to infer ship-scale drag outcomes. Common ways of measuring hydrodynamic drag are to use towing tanks, flow cells and rotating cylinders or discs. The 26^th^ International Towing Tank Conference [ITTC] (ITTC TRC 2011) compared the practicality of various methods for measuring skin friction for rough surfaces in the marine sector and concluded that using flat plates in towing tanks offers the best combination of accuracy and complexity. Towing tank tests are, however, large scale enterprises that are time-consuming and expensive to carry out, and speeds are usually limited to less than 10 knots. Rotating discs offer practical advantages over towing tank tests since they can rapidly reach high peripheral velocities and their rotational speed is easily varied without having to handle large volumes of water.

The minidisc method presented here is proposed as a route for rapid evaluation of coating technologies that is complementary to established microfouling assessment methods. With the minidisc method, rankings are based on an experimental drag metric rather than a simple slime metric that is assumed to be a proxy for drag. In the best tradition of marine fouling bioassays, this method is based on small scale test pieces that can be rapidly fouled and measured. The aim was to develop a method that is experimentally simple yet has high precision. This was achieved by using an analytical rheometer which is operated by a single person on an open laboratory bench. The torque on rotating discs, expressed as a moment coefficient (*C_m_*), can be used directly to measure the relative drag due to surface roughness. In addition to simple torque screening of coatings, the minidisc drag data can be further processed for comparison with the available scientific literature and accumulated knowledge of decades of drag research using a variety of methods of data acquisition and mathematical treatment. This is accomplished *via* roughness function analysis.

## Materials and methods

### Measurement system

Rheometer discs with diameter 40 mm and thickness 2 mm were machined from white polyacetal plastic rod (Delrin^TM^, Du Pont). This polymer was selected because it has good dimensional stability and low water uptake (<1%) at saturation (Sdplastics.com 2020).

**Figure 1.**
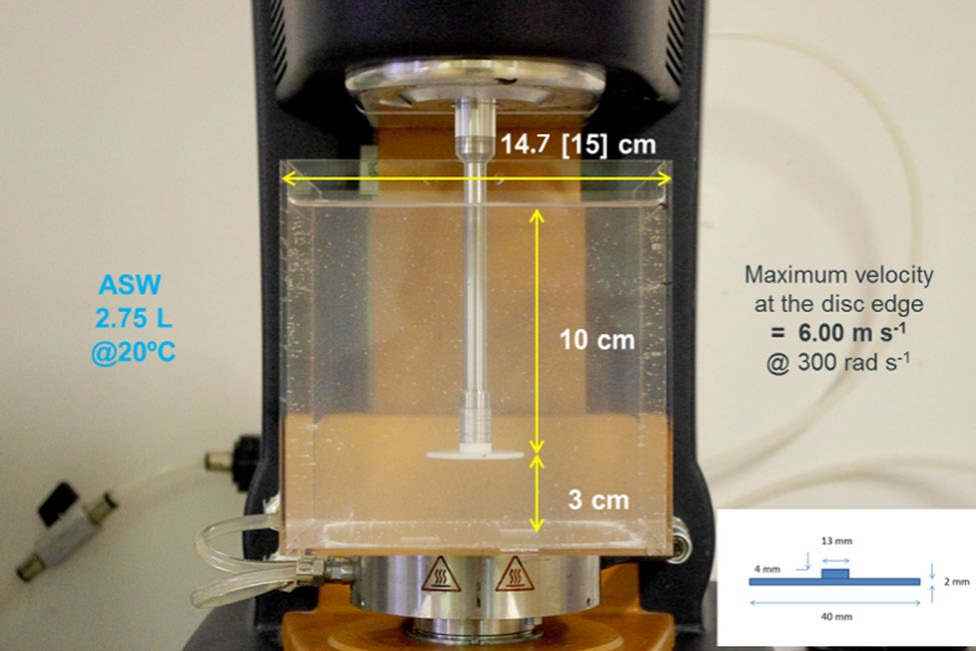
Experimental apparatus: rheometer with temperature-controlled water reservoir.

The torque acting on the discs rotating in a water reservoir was measured by attaching them to the shaft of an analytical precision rheometer (DHR-2, TA Instruments) with torque resolution of 0.1 nN m^−1^. The water reservoir was cubic with sides of transparent acrylic plastic having external edge length 150 mm (147 mm internal) (Figure 1). This size of reservoir is the largest that can be accommodated on the rheometer platen. Initial use of an open cylindrical container (glass beaker) resulted in the water being set in rapid circular motion by the rotating disc, forming an increasingly deep vortex around the shaft as the speed increased, which resulted in the water overflowing the container even at moderate speed. The square cross-section of the cubic tank prevented this, and maintained a relatively smooth water surface. Sodium chloride solution (3.5 wt%) was used to maintain approximately the same osmotic pressure and kinematic viscosity as seawater. The base of the reservoir consisted of a flat stainless steel plate machined to fit closely on to the thermostated platen of the rheometer to allow accurate temperature control of the water at 20 °C ± 0.1 °C. Maintaining a constant water temperature is important owing to the strong temperature dependence of the viscosity of the water, which impacts the torque acting on the disc.

Discs were screwed onto a threaded adapter on the rheometer shaft with the test side of each disc facing downwards at 3 cm above and parallel to the bottom of the tank. A disc rotating close to a fixed surface may be subject to a net downward force, due to the reduced pressure resulting from radial expulsion of fluid in the narrow gap below the disc (Peters 1967). To avoid this effect, the distance of the disc from the bottom of the tank was determined empirically during a control run with a blank disc by minimising the magnitude of the normal force acting on the disc surface, a parameter which is recorded by the rheometer. To record the torque response of a disc, the rotational velocity was increased from 30 rad s^−1^ up to 300 rad s^−1^ (maximum velocity) at increments of 15 rad s^−1^ with the speed held constant for 10 s at each speed. Disc rotation was stopped at the maximum velocity and after 60 s at rest the speed ramp up was repeated for control and sandpaper discs.

### Surface roughness measurements

Two surface roughness measurement instruments were used, each of which provides multiple surface roughness parameters simultaneously: (1) Blue Light Interferometer (MikroCAD, LMI Technologies, Germany). Measurement area 26 mm × 26 mm, cut-off wavelength 10 mm. (2) Optical surface profiling *via* Alicona Infinite Focus Microscope (Alicona GmbH, Austria).

The area imaged is defined by objective power (5×, 10× or 20×). The relevant surface roughness parameters are the arithmetical mean height (Sa) and the maximum peak-to-trough height (Sz) for the defined area. The corresponding parameters for a linear surface profile between two points are Ra and Rt. The correlation between Sa values obtained by the two different measuring instruments was:

$${\text{Sa}}_{\text{ALICONA}}(\mu m)\;=\;0.{882\text{ x }\text{Sa}}_{\text{BLUE}\;\text{LIGHT}}\;\;\left(\mu m\right)\;\;\;r^{2}=\;0.992$$

### Marine coatings

Four commercial marine coatings produced by AkzoNobel (UK) were applied by spray to the front faces only of discs. Each coating layer was applied with an average dry thickness of ∼100 µm −150 µm.

The coating types were: (1) ACP (Control): Intershield® 300, a hard anticorrosive paint with no anticipated fouling control properties; (2) FRC: Intersleek® 700, a first-generation silicone foul release coating; (3) CDP: Interspeed® 6200, a TBT free, controlled depletion polymer self-polishing antifouling system; (4) SPC: Intersmooth® 7460HS SPC, a TBT free, self-polishing copolymer antifouling.

### Control discs

Duplicate Delrin discs coated with the ACP control gave similar torque responses, with a mean standard deviation of 1.1% (n = 24) over the whole rotational velocity range. The mean corrected one-face torque value (*M_1_* Blank) of these discs acts as the control for the fouling exposure trials.

### Marine fouling exposure

**Figure 2.**
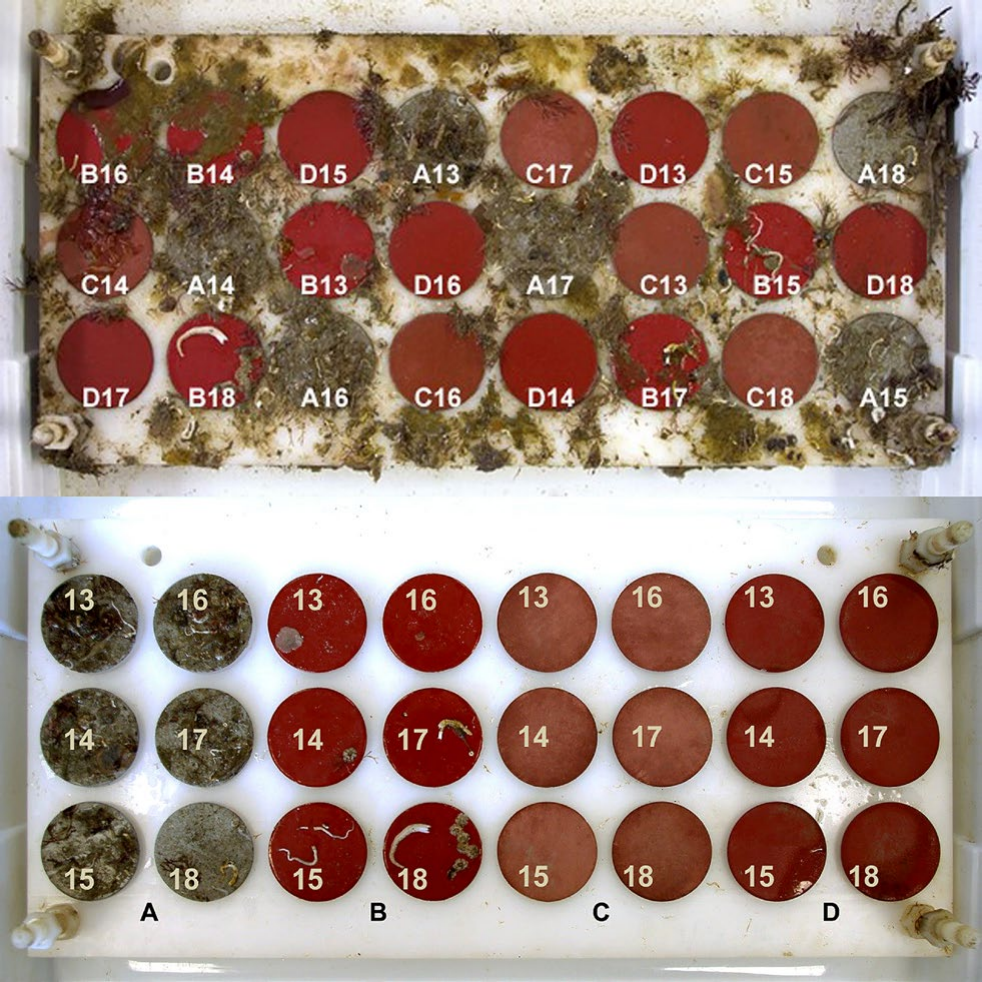
Sample board with discs fouled after 176 days exposure. Above: As removed from the sea, discs in random placement. Below: After rheometer testing, discs replaced in numbered order. A = ACP, B = FRP, C = CDP, D = SPC

After drying/curing of the applied coatings, the surface roughness was measured by blue light interferometry (S1.1) and the torque of all discs was measured using the rheometer. The coated discs were attached to four submersible Delrin sample boards in machined recesses which held the disc faces flush with the board surface (Figure 2). Each sample board bearing six duplicates of all four coatings (total 24 discs) was exposed to fouling in the sea for a period of up to 176 days between February and August 2016. Discs on each of the four backboards were arranged randomly to eliminate spatial effects. The boards were immersed from a floating pontoon at the National Oceanography Centre Southampton UK (NOCS), location 50°53′30″N, 1°23′40″W, in an estuarine environment facing SW with the top of the board at a depth of 0.5 m. Polypropylene netting (1 cm mesh size) was fitted over each board at a height of ∼3 cm above the surface and extending 10 cm beyond the edges to prevent fish grazing on the slime. At the end of the relevant immersion period one sample board was permanently removed from the sea and placed in a tightly closed plastic box for transport to rheometer testing the same day. The discs were kept wet over a reservoir of seawater (but not immersed, as movement of the water during transport could wash away fouling). For testing, discs were carefully unscrewed from the sample board taking care not to touch the fouled faces, and the disc perimeter was wiped clean to remove any microfouling which might have formed.

### Sandpaper surfaces

A series of artificial surfaces with known roughness was prepared by covering disc surfaces with different grades of self-adhesive sandpaper (Draper Tools). The grit size of the sandpapers accords with Federation of European Producers of Abrasives [FEPA] standards (S2). Sandpaper discs 40 mm in diameter were laser-cut to ensure exact dimensions and a smooth periphery and affixed to the face side only of discs. The added thickness and mass of sandpaper discs or coatings was not considered in calculations. Surface roughness profiles were measured by focus variation microscope (Alicona, GmbH, Austria) and reported as the mean of 10 determinations made at random points on the disc, and by blue light interferometry using a MikroCAD (LMI Technologies, Germany) interferometer (S3). The surface roughness parameter chosen by Schultz and Flack (2003) for roughness function calculations was the maximum peak to trough roughness height of a linear profile (*Rt*) × 0.75. In this work the equivalent maximum peak to trough surface height of an area (*Sz*) was used in place of *Rt*.

### Calculation

The moment or torque coefficient (*C_m_*) derived from both sides of a rotating disc is a dimensionless number defined by Granville (1978, 1982) as:

(1) $$C_{m}=\frac{4M}{\rho \;r^{5}\;(\varphi {\omega )}^{2}}$$

where *M* is the moment or torque acting on one side of the rotating disc. Note that other authors have used the notation *C_m_* to represent the momentum coefficient for one side of a disc only, e.g. Childs (2011) or have used the notation *M* for the torque acting on both sides of the disc (Schultz and Myers 2003). For clarity, the present calculations will use subscripts to denote the number of disc sides each parameter refers to, thus:

*M_1_* Torque acting on one side of a rotating disc; *M_2_* Torque acting on both sides of a rotating disc; *C_m1_* Moment or torque coefficient for one side of a rotating disc;

*C_m2_*  Moment or torque coefficient for two sides of a rotating disc

For a disc rotating in a constrained volume of fluid, a mean tangential swirling or vortex flow of the fluid will be induced in the direction of rotation, especially in cylindrical containers, which reduces the effective angular velocity of the disc relative to the fluid. The ‘swirl factor’ φ may be determined experimentally if the disc can be operated in a large unbounded volume of water (Granville 1982). In the configuration used by Granville and by Schultz and Myers (2003) with a large disc rotating in a closed cylindrical reservoir, φ was experimentally determined to be 0.854. In a smaller tank as used here the value of φ is predicted to be lower (MP Schultz 2020 personal communication, unreferenced) as the small volume of water and the proximity of the disc to the tank bottom and walls will promote more vigorous agitation and greater co-rotation of the water. The hydrodynamic flow pattern induced by the rotating disc has not been determined, but there was clearly co-rotation of the water which was visible when the disc rotation was suddenly stopped at the maximum velocity. The value of φ could not be experimentally determined, as the fixed construction of the rheometer and the limited size of the platen makes it impossible to re-position the disc in a larger reservoir to make the necessary comparison. Instead a value of φ was assigned that caused the disc sandpaper roughness functions to collapse onto a standard Nikuradse-type sand curve.

Mathematical derivations of *C_m_* leading to Equation 1 assume that edge effects, i.e. the torque contribution of the disc edge, are negligible (Childs 2011). In practice, the edge of discs will generate significant drag, which depends on their thickness, as the perimeter of the disc is moving at maximum velocity. Nelka (1973) quotes a formula from Dorfman (1963) which allows the moment coefficient (and hence torque) due to the disc edge to be estimated:

(2) $$C_{m}=C_{m}^{'}/(1+2.5\;\frac{b}{r})$$

where in Dorfman’s notation C’_m_ is moment coefficient due to the whole disc (front face, back face and edge), C_m_ is moment coefficient due to front and back disc faces only, b is disc thickness and r is disc radius.

Substituting the present disc dimensions of b = 2 mm, r = 20 mm in Equation 2

(3) $$\frac{C_{m}}{{C'}_{m}}=1/(1+2.5\;\frac{2}{20})=1/1.25=0.8$$

Equation 3 predicts that the front and back faces of a clean unfouled blank minidisc together contribute a fraction of 0.8 (80%) of the whole disc torque, so half this value (40%) can be assigned to each face, while the edge contributes the remaining 20%. The rear disc face and disc edge combined therefore give rise to (40% + 20%) = 60% of the torque of the whole blank disc, so that an estimate of the torque *M_1_* due to the front face alone of an experimental disc can be made. This is calculated by subtracting the torque acting on the rotating shaft alone, plus 60% of the torque of a blank control disc, from the raw torque data for any disc condition over the whole velocity regime.

If *M_x_* = Torque for whole experimental geometry (disc plus shaft), *M_s_* = Torque for shaft alone, and *M_b_* = Torque for whole blank disc

(4) $$M_{1}=\;M_{x}-M_{s}-0.6M_{b}$$

Rewriting and rearranging Equation 1

(5) $$M_{1}=\frac{C_{m2}\rho r^{5}\varphi^{2}}{4}\omega^{2}$$

Density of 3.5% NaCl solution at 20 °C, ρ = 1025 kg m^−3^

A plot of *M_1_* vs ω^2^ has slope ($C_{m2}\rho r^{5}\varphi^{2})/4$so Cm2 ρr5φ2)/4 that a value for *C_m2_* can be obtained from the value of the slope at a specific velocity.

The rotational Reynolds Number (*Re_r_*), for a rotating disc is given by Equation 6 (Niebles Atencio and Chernoray 2019):

(6) $${Re}_{r}=\frac{(\varphi \omega )r^{2}}{\nu }$$

where: fluid kinematic viscosity (ν) is 1.05 × 10^−6^ m^2^ s^−1^ for seawater at a salinity of 35 g kg^−1^ and a temperature of 20 °C (Kaye and Laby 1995).

The range of rotational Reynolds number (*Re_r_*) this apparatus operates over therefore depends on the swirl factor, the maximum attainable with no co-rotation of the water being 1.14 × 10^5^ when φ = 1.

#### Similarity law analysis

The roughness functions for the rotating disk surfaces were calculated using the similarity law analysis developed by Granville (1978, 1982). The roughness function is the magnitude of the downward shift in the logarithmic region of the mean velocity profile of the boundary layer that is caused by surface roughness (Schultz and Myers 2003). Granville’s procedure involves finding the difference in the C*_m_* values of smooth and rough disks at the same value of *Re_r_* √*C_m_* and calculating the roughness function (*ΔU^+^*) and roughness Reynolds number (*k*^+^), which is the ratio of the roughness length scale to the viscous length scale, through an iterative solution of Equation 7 and 8. Using the notation in Schultz and Myers (2003) where *R* = radius, *k* = surface roughness and *κ* (kappa) = von Karman constant, Granville’s equations are:

(7) $$k^{+}=(\frac{k}{R})\sqrt{\frac{5}{8\pi }\;}{Re}_{R}\sqrt{C_{m}}\{1-[\frac{2}{\kappa }-{\Delta U}^{+'}]{\left(\sqrt{\frac{C_{m}}{40\pi }}\right)}_{R}\}$$

(8) $${\Delta U}^{+}=\sqrt{\frac{8\pi }{5}}[{\left(\frac{1}{\sqrt{C_{m}}}\right)}_{S}-{\left(\frac{1}{\sqrt{C_{m}}}\right)}_{R}]+\frac{1}{5}{\Delta U}^{+'}$$

Plotting *ΔU^+^* against log *(k^+^)* facilitates comparison with other published roughness function results such as the uniform sand curve of Nikuradse (1950), who established that frictional resistance, and hence the roughness function, varies according to the magnitude of *k^+^*. At low *k^+^* the thickness of the laminar boundary layer is still greater than the surface roughness projections, and roughness does not affect resistance. As *k^+^* increases the boundary layer thickness is reduced so that individual roughness elements extend through the viscous laminar sublayer and cause vortices that result in a loss of energy. At high enough *k^+^* the thickness of the boundary layer is so small that all the roughness elements project through it, giving a constant drag contribution. Thereafter in the fully rough flow regime the resistance becomes independent of *k^+^* and depends only on the relative roughness.

The uniform sand curve was not directly plotted by Nikuradse (1950) and must be constructed from his flow data. It has been noted (Niebles Atencio and Chernoray 2019) that there is no established agreement in the literature on a ‘true’ Nikuradse roughness function owing to the spread of the original data. Grigson (1984) plotted an improved sand roughness function curve after re-evaluating and correcting the original experimental data of Nikuradse, and comments on the excellence of the data.

Various authors have developed expressions for uniformly distributed roughness functions and compared them with Nikuradse’s data. A standard curve can be constructed from a set of generic roughness function equations such as those given by Cebeci and Bradshaw (1977) as noted by Andersson et al. (2020). According to Hunsucker (2016), Ligrani and Moffat (1986) gives an expression for the Nikuradse-type roughness function which takes the form:

(9) $$\Delta U^{+}=[(\frac{1}{\kappa })\ln k_{s}^{+-}-B_{N}+B_{1}]\;\text{sin}\;(\frac{\pi }{2}G)$$

where B_N_ and B_1_ are taken as ∼ 8.5 and 5.0 respectively, and where the value of G depends on the flow regime. For hydraulically smooth surface flow G = 0, and for fully rough flow G = 1. The transitional rough flow regime is taken to occur over the range 3<${\;k}_{s}^{+}$<70 (where $k_{s}^{+}$ is the uniform sand roughness Reynold’s number). Other authors’ range limits vary slightly from these values (Demirel et al. 2017) without significantly affecting the overall shape of the curve. In this transitional flow region, taking the limits as k^+^_smooth_ = 3 and k^+^_rough_ = 70:

(10) G=ln(ks+ksmooth+)ln(krough+ksmooth+)

Equation 9 with the above values for G is used to generate the reference Nikuradse-type curve used in this analysis.

Colebrook (1939) investigated irregular surface roughness such as that of corroded metal pipes and derived a theoretical formula for flow in the transitional region between smooth and fully rough flow regimes that differs from Nikuradse’s results. Colebrook’s formula, most commonly used in the form of Equation 11 (Andersson et al. 2020), applies to all flow conditions:

(11) $$\Delta U^{+}=(\frac{1}{\kappa })ln(1+k^{+})$$

Both the graph of Colebrook’s monotonic expression and Nikuradse’s uniform sand curve have the same slope in the fully rough regime (*k^+^* > approx. 70) with *ΔU^+^* approaching zero in laminar flow (*k^+^* < approx. 3) but differ in form in the transitional region.

## Results

### Sandpaper calibration

**Figure 3.**
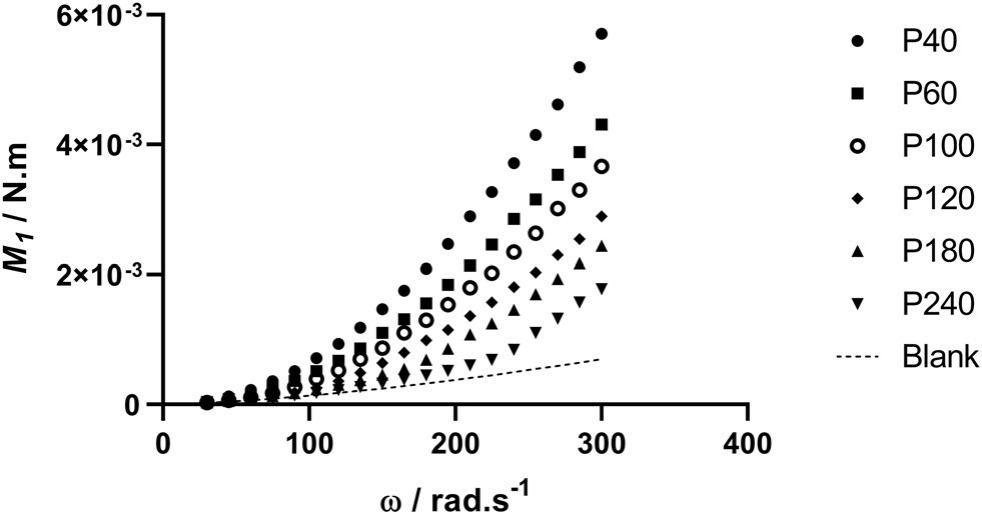
Torque developed on one face of sandpaper covered discs *vs* rotational velocity.

The torque on sandpaper covered discs increased with increasing roughness grade (Figure 3).

**Figure 4.**
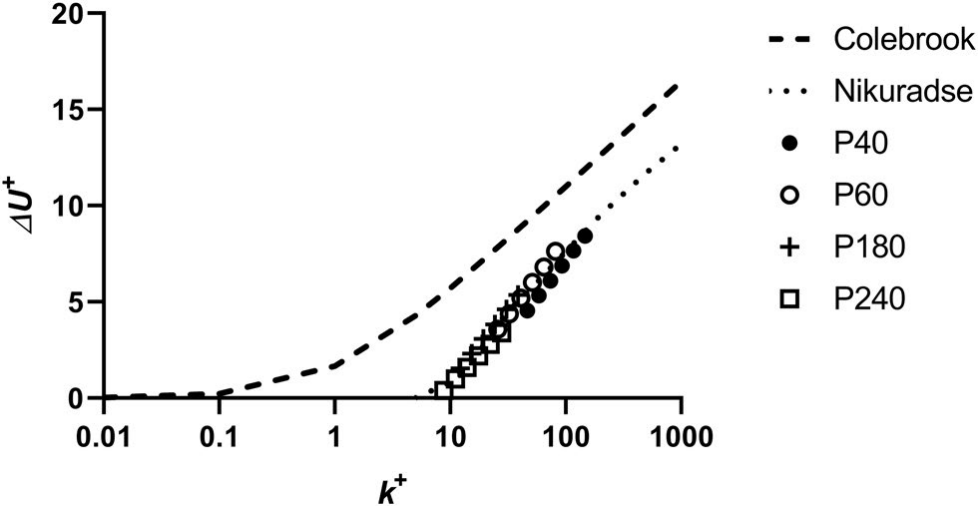
Roughness functions for sandpaper discs.

Even the coarsest grade 40 discs induced only moderate co-rotation of the water around the shaft axis. In cylindrical tanks, the secondary co-rotational flow that is set up in the tank due to the steady state rotation of the disk is accounted for by the swirl factor φ. If this cannot be experimentally determined, as in the present case, a suitable value for φ may be allocated by inspection of the roughness function data, such that these may be shifted to higher or lower roughness Reynolds number (*k^+^*) to collapse onto an established curve such as that of Nikuradse (Schultz MP, private communication, not referenced). Choosing the appropriate roughness length scale is an additional factor that interacts with φ in obtaining the appropriate *k^+^* (Flack and Schultz 2014). The factor of 0.75 × peak-to-trough roughness suggested by Schultz and Flack (2003) as suitable for sand grain rough surfaces was used for the sandpaper calculations, but taking the area-based peak to trough roughness (*Sz*) rather than their linear value *Rt*. Flack and Schultz (2014) suggested that a different scale or combination of scales is likely required in each flow regime. With this roughness length scale fixed, setting the value of φ = 0.60 collapsed all the sandpaper data onto the calculated Nikuradse-type curve. Roughness functions for four of the sandpaper discs are shown in Figure 4, the other sandpaper grades fall in between these values.

This value of 0.60 for φ was accordingly used for all calculations, although it may not be appropriate for disc roughness that is outside the range of the sandpaper grades or is irregular, as may be the case with macrofouling organisms. In the latter case a different swirl factor may be required.

### Coatings

The coatings represented four different product types: an anti-corrosive paint (ACP) that served as an inert control, a biocide-free silicone foul release coating (FRC), a controlled depletion polymer biocidal (CDP) and a self-polishing copolymer biocidal coating (SPC). Freshly coated discs had overall average *Sa* = 5.3 µm (S4.1) which is smoother than the uncoated polymer discs (*Sa* = 8.0 µm). Optical microscopy revealed some concentric groove patterns on these machined polymer disc faces which likely account for the observed surface roughness. When relatively thick polymer coatings are applied these patterns will be covered up and the dried coatings produce a smoother surface. Torque for the coated discs were all within ±2% of the overall mean, except for the FRC-coated discs which had a marginally higher torque (106% of the mean) over the whole velocity range, corresponding to a slightly rougher initial surface condition. This suggests that the quality of application of the FRC coating to the disc may have been sub-optimal, as FRC coatings typically have lower surface roughness than CDP and SPC coatings (Stenson et al. 2013). Waviness of the FRC surface is not likely to be a consideration in rotational motion.

During the static exposure period, each board was visually inspected at 2-week intervals and a much greater degree of fouling was observed on the inert coating throughout. After 110 days the protective polypropylene netting had heavy fouling attached, as did the surface of the Delrin sample holder board. Discs coated with ACP and FRC had accumulated some hard-shelled fouling organisms (barnacles, tubeworms) while discs coated with biocidal coatings CDP and SPC had not.

The protective netting on the board exposed for 176 days became completely obstructed by heavy fouling, and when the fouled netting was removed the board beneath appeared cleaner than the board immersed for 110 days (Figure 2). While the polypropylene netting did prevent large fish from gaining access to the test surfaces, small fish (blenny, *Lipophrys pholis*) and crabs were found beneath the netting and these predators could have removed some of the accumulated biofouling in the relatively sheltered environment created by the heavily fouled netting.

**Figure 5.**
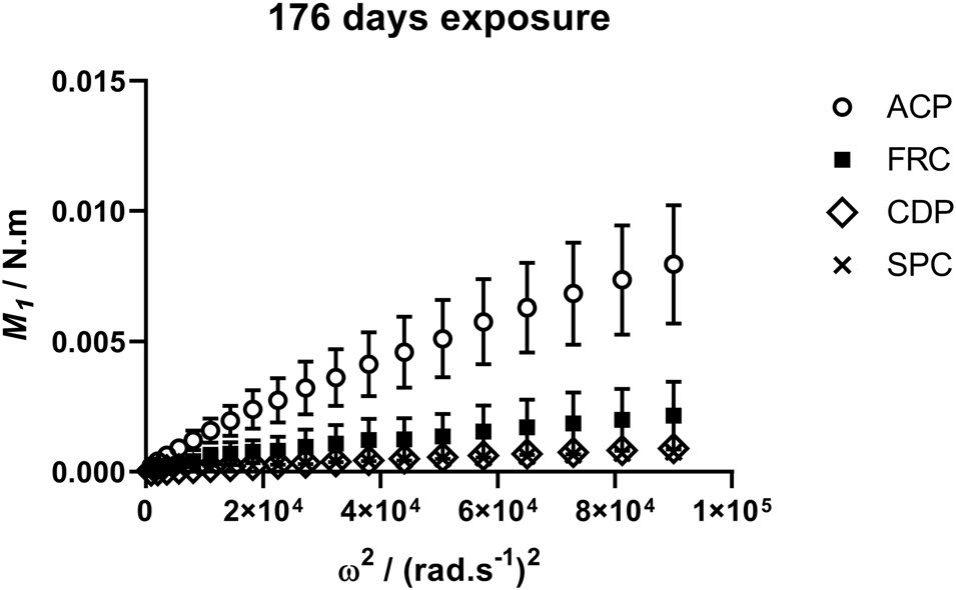
Torque developed on fouled coated disc faces at the end of the exposure period. Error bars are ± SEM (n = 6).

**Figure 6.**
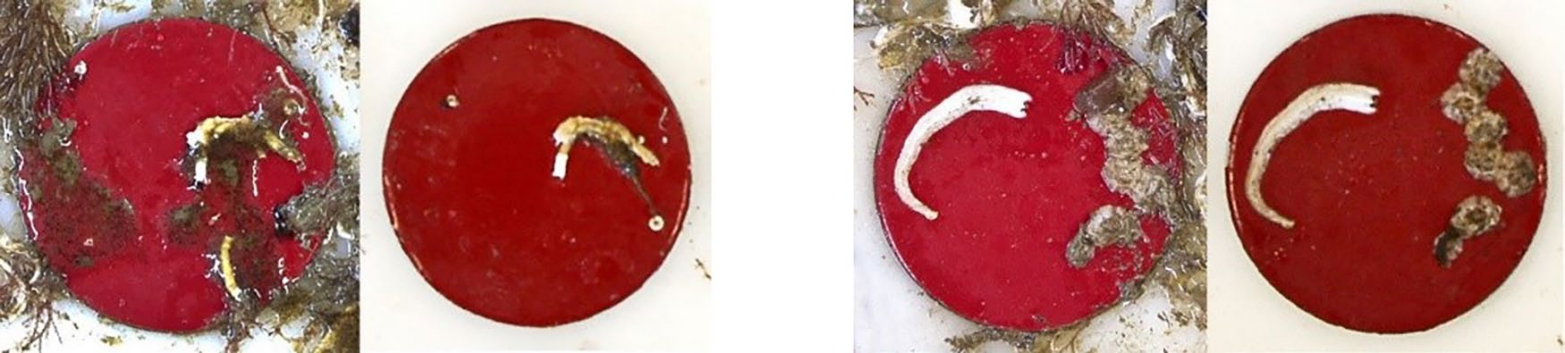
Two FRC coated discs after 176 days fouling. Disc B17 (Left image pair), Disc B18 (Right image pair). Lefthand side image of each pair is *before* rheometer testing, and righthand side image is *after* rheometer testing.

**Figure 7.**
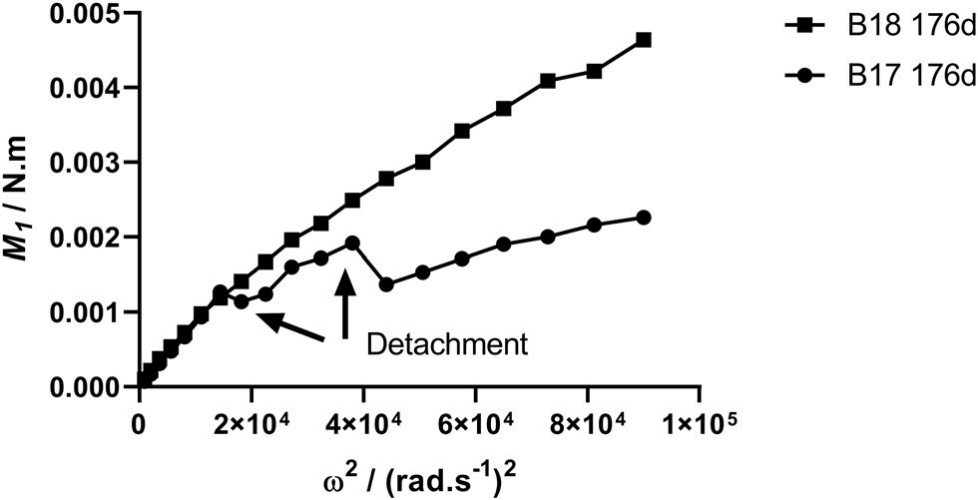
Torque response of two replicate fouled FRC discs, showing detachment of macrofouling organisms.

The measured torque for each disc coating type reflected the visible amount of fouling coverage, with the inert anticorrosive-coated discs having considerably higher torque throughout the whole exposure period than the fouling control coated discs. Torque values for duplicates of the SPC coated discs were all low and similar. There was a statistically significant difference (*p* << 0.05) between the raw torque values *M_1_* (for one disc face) at maximum rotational velocity for the fouled coatings from 85 days of exposure onwards as determined by one-way ANOVA (S5). The *M_1_* responses after 176 days, averaged over all discs for each coating type, are shown in Figure 5:

Up to 85 days exposure, *M_1_* for all fouled discs remained below the maximum torque recorded for the roughest sandpaper P40 grade (*M_1_* = 0.0057 N m^−1^ at 300 rad s^−1^), while torque for the SPC coated discs remained below this value even after the longest exposure period. Macrofouling was visible on the ACP and FRC discs from 85 days exposure onwards. Torque varied greatly between heavily fouled duplicate discs, due to the natural spatial variation in fouling coverage as seen in Figure 2. Some discs shed loosely attached fouling when rotated, resulting in abrupt decreases in torque. This was observed mainly with the FRC coating which is designed to release fouling organisms under flow conditions. Images of two FRC-coated discs taken before and after testing (Figure 6) show that while no fouling organisms became detached from one of them (disc B18) a significant tubeworm casing and some minor peripheral fouling were lost from a similarly fouled duplicate (disc B17), giving rise to the step-wise decreases in torque observed (Figure 7).

**Figure 8.**
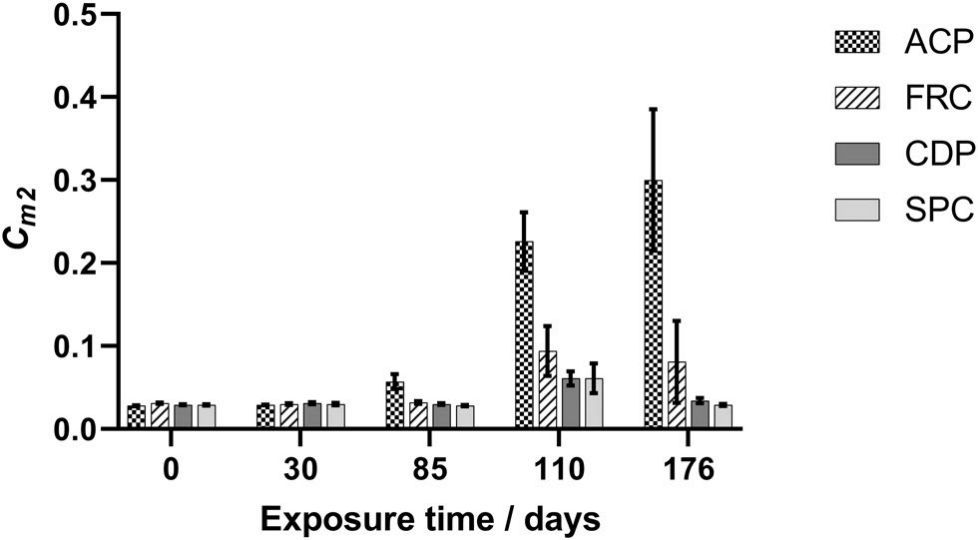
Torque of fouled discs during the exposure period, divided into coating types. Error bars are ± SEM (n = 6).

The coefficient of momentum *C_m2_* at the maximum rotational velocity was obtained for all coated discs throughout the exposure period and the average *C_m2_* values for each coating set are compared in Figure 8. After 176 days discs coated with FRC had 73% lower *C_m2_* and discs coated with CDP and SPC 89% and 90% respectively lower, than discs coated with the inert control ACP.

**Figure 9.**
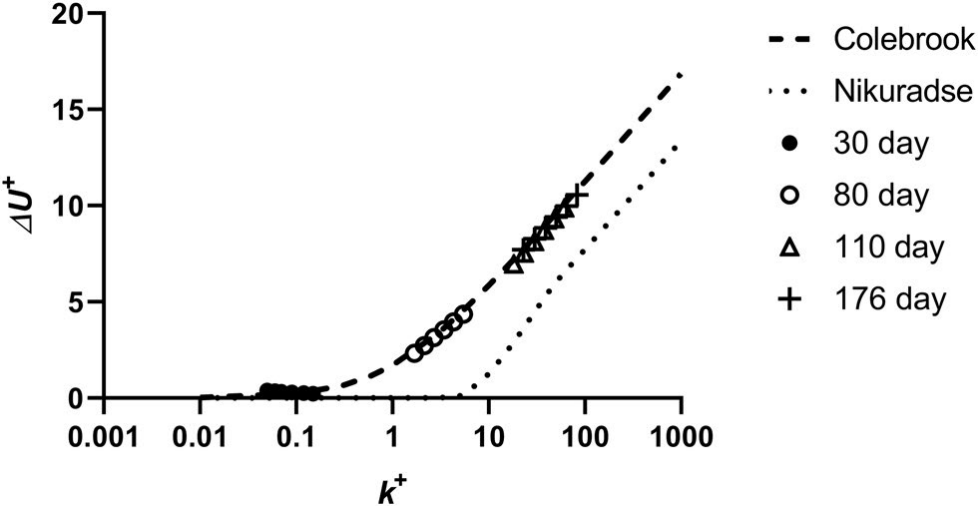
Roughness functions for fouled ACP discs over time.

Roughness functions for the fouled discs gave better fits to the Colebrook irregular roughness function, especially at low *k^+^*, than to the Nikuradse-type uniform sand curve. Maintaining the value of φ = 0.60, each fouled disc roughness function could be displaced along the *k^+^* abcissa to collapse onto the Colebrook curve by assigning a suitable surface roughness height. The resulting roughness functions for ACP (the most heavily macrofouled coating) over the exposure period are shown in Figure 9.

**Figure 10.**
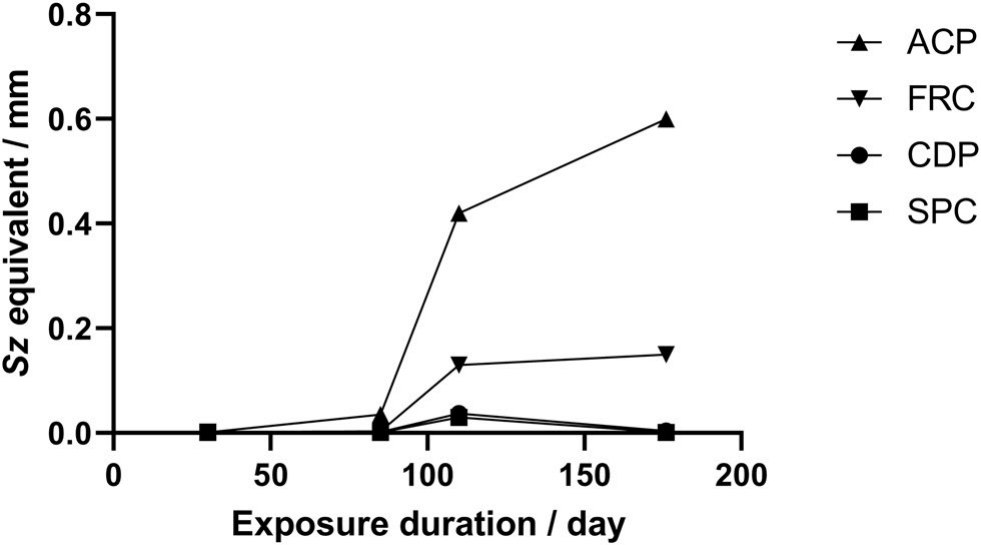
Roughness height needed to align fouled disc roughness functions with Colebrook’s irregular roughness data.

The roughness heights required to bring the roughness functions for all the fouled disc exposures into line with the Colebrook function are given in Table S5.1 and plotted in Figure 10.

## Discussion

The primary function of FC coatings is to reduce fouling accumulation on ships’ hulls and thereby decrease the drag experienced when vessels are underway. Developing FC coatings requires extensive testing, and for coating companies down-selection of candidate coatings is a critical process that necessarily focuses costly and limited R&D resources on the most promising formulations and technologies. Simple and reliable test methods are therefore of great interest. Industry and academia use various down-selection routes to directly assess FC coating “slime efficacy”, such as bioassays (Ramsden and Longyear 2014; Briand 2009). Bioassays provide the greatest advantage when they are simple to perform and compress fouling time scales by being rapid to execute, goals commonly achieved by using small test pieces such as microscope slides, which can be deployed in very large parallel experiments with logistical ease. Often the measure of “efficacy” is some metric of total slime load (e.g. percentage coverage, biofilm roughness). From a ship owner’s perspective, however, some economic aspect of ship operational efficiency related to drag, such as projected fuel oil consumption, is a more relevant metric. This requires experimental measurement of hydrodynamic drag using accessible test surfaces in towing tank or rotating disc systems.

Rotating discs offer practical advantages over towing tank tests since they are much smaller and can rapidly reach higher peripheral velocities. The resisting torque is measured with an in-line torque meter (Holm et al. 2004). Typically disc diameters of ∼30 cm have been used to provide a large enough surface to achieve the sensitivity to detect increases in torque due to fouling, and to achieve high rotational Reynolds numbers (*Re_r_*) at the disc edge, approaching the range of that experienced by ships as they move through the water.

Various authors have studied the drag increase due to marine biofilms using rotating discs of this size. Loeb (1981) and Loeb et al. (1984) studied the influence of microbial fouling films on the hydrodynamic drag of rotating discs and showed that drag increases of 10% to 20% could be caused by microbial slimes, while Holm et al. (2004) found microfouling drag penalties ranging from 9% to 29%. Yeginbayeva et al. (2019) used a rotating disc device to assess fouling on 30 cm diameter discs coated on one side with various commercial coatings and either exposed in the sea on the west coast of Sweden or fouled in the laboratory. Tenacious slime and algal fouling remaining on discs after initial dynamic testing resulted in from 28% to 41% increased drag in the range of test speeds 13 knots to 26 knots. However, discs this size require correspondingly large tank volumes and can still be cumbersome to work with. Marine exposure and transport of these discs from an immersion site to a testing rig can be problematic and exchanging discs between tests and establishing equilibrium flow is relatively time consuming. In addition, they are not suitable for testing of expensive or exotic surface modifications because of their large surface area.

Previously the feasibility of measuring the increase in torque caused by marine biofilm fouling on small (2.5 cm diameter, 8 mm thick) test pieces coated with polymethyl methacrylate (PMMA) using a precision rheometer to serve as a high sensitivity (nN m^−1^) torque monitor has been reported (Dennington et al. 2015). The motivation for the present study was to refine the technique to assess the feasibility of using this method as a rapid screening tool for identifying the potential of marine coating formulations or surface modifications to reduce biofilm fouling and the associated hydrodynamic drag. It was hypothesized that this rapid and simple benchtop method would be sensitive enough to detect the progression of early fouling development as well as differences between the performance of anti-corrosion paint and coatings specifically formulated to reduce fouling. To test this hypothesis, the ability of the method to differentiate the drag caused by various rigid roughness elements was determined by making measurements on “minidiscs” coated with sandpapers of defined roughness. Measurements were then made on minidiscs coated with various anticorrosion and AF coatings after various times of seawater immersion for up to 176 days.

At the maximum rotational speed of 300 rad s^−1^ (2,865 rpm) the peripheral velocity of the minidiscs is 6 m s^−1^ (11.7 knots), which is similar to that in a towing tank and approximates the current average speed for a merchant ship which has fallen from 15 knots in 2010 (Moritz Bollmann et al. 2010) by around 25% (Paris 2019). The Reynolds number for a full-scale vessel would typically be 5 × 10^8^ − 5 × 10^9^ with that for a towing tank model one to two orders of magnitude less (Shen and Hughes, Shen et al. 2015). The theoretical maximum *Re_r_* achievable with the rheometer apparatus is 1.14 × 10^5^, but co-rotation of the water in the cubic reservoir reduces this to 6.81 × 10^4^. For a hydrodynamically smooth disc, applying Granville’s analysis (Granville 1973) yields a corresponding wall shear stress of 24 Pa (S4). The shear stress for an average ship or a flat plate of 100 m is about 50 Pa (Yeginbayeva et al. 2019). Therefore, the rotational (tangential) velocity and the streamlined velocity in towing tanks are not directly comparable.

In the present study coated discs that were immersed in the estuarine environment became covered with fouling over the summer exposure period. Biofilm alone succeeded in colonising the discs coated with the biocidal CDP and SPC, while some larger macrofouling organisms (barnacles and tube worms) became attached to discs coated with ACP and FRC. Duplicate discs bearing the same coating type showed varying levels of fouling, as is expected in biological exposures, but averaging the torque of these gave significant results for coating performance. The relative degree of fouling was indicated by the magnitude of the torque developed on rotation, expressed *via* the raw torque or the momentum coefficient. Detachment of fouling organisms from the FRC was revealed by sudden drops in torque as the rotational speed increased. Such events facilitate an estimate of the tenaciousness of fouling attachment, but they cause difficulty in determining the *C_m_* at higher velocity had the fouling not detached. Clear differences between coatings were apparent from 85 days exposure onwards, with the heavily fouled anticorrosion coating giving *C_m_* values up to 10x higher than the fouling control coatings. The magnitude of this parameter may therefore serve as a simple relative measure of drag due to fouling when comparing coatings.

The well-characterised disc geometry allows further analysis to be attempted. The rheometer torque response was calibrated using sandpaper surfaces. Applying similarity law analysis as developed by Granville (1978, 1982) allowed roughness functions for the test surfaces to be estimated. The sandpaper roughness functions could be collapsed on to a standard Nikuradse-type uniform sand curve by adjusting the magnitude of either the chosen surface roughness parameter or the value of φ, both of which move the *ΔU^+^* curves to higher or lower *k^+^* values along the abscissa. With the surface roughness parameter fixed at 0.75 × Sz, the value of φ was changed to bring the *ΔU^+^* data in line with the uniform sand curve. The value of φ = 0.60 which collapsed the data for all the sandpaper grades onto the uniform sand curve was used for all subsequent calculations. This is necessarily an approximation, as the degree of co-rotation of the water may vary depending on the height and regularity of fouling roughness elements. However, the good agreement of the sandpaper results with standard sand grain roughness data supports the validity of the minidisc measurement system for enabling further hydrodynamic analysis, at least for uniform surface roughness textures.

The roughness functions of fouled discs showed only poor to moderate agreement with the Nikuradse-type sand curve. Schultz and Myers (2003) pointed out that the form of the roughness function is not universal for all roughness types, and that most naturally occurring rough surfaces do not behave like closely packed uniform sand grains. Roughness functions for the fouled discs could instead be collapsed onto the Colebrook equation for irregular surface roughness by assigning appropriate values for roughness height.

The spatial distribution of larger fouling organisms is uneven, which will affect their contribution to the overall torque of these small disc surfaces. The influence of individual roughness elements increases with the distance from the disc centre, as an organism attached near the spinning disc periphery will move faster and exert a greater moment than if it were attached at the centre of the disc. The test method will therefore become numerically less accurate as uneven macrofouling coverage develops on the disc surface. The criteria for validity of the wall similarity hypothesis are that the Reynolds number is sufficiently high and the roughness is small compared with the boundary-layer thickness (Schultz 2007), conditions which again may not apply for discs bearing macrofouling organisms such as barnacles, making these results questionable. Nevertheless, the good fit of the fouled surfaces’ roughness functions to the Colebrook function for irregular surface roughness suggests that with further certainty regarding the swirl factor and its effect on roughness function, the data may be scalable and potentially of use in estimating ship-scale drag. To further validate the utility of the method as a rapid screen for predicting the AF potential of coatings or other surface modifications direct comparisons of different coatings should be made with the more established techniques.

## Conclusions

Rheometry is an experimentally simple means of quantifying drag due to surface roughness on minidiscs. It is rapid to perform, making it suitable for high throughput testing of coated surfaces. The use of polymer discs with a high radius to thickness ratio maximises the sensitivity of the torque response to the condition of the disc face. The torque resulting from the fouled face alone is used to obtain an estimate of the equivalent coefficient of momentum for a double-sided disc (*C_m2_*). The high sensitivity of the analytical rheometer enables very small differences in roughness for the as-applied coatings to be detected, and can easily differentiate between statically grown fouling accumulated on different commercial fouling control coatings exposed for varying periods in a marine estuary. Directly comparing torque values, quantified as the *C_m2_*, permits rapid evaluation of relative fouling control performance. After 176 days exposure to fouling, discs coated with FRC had *C_m2_* values 73% lower, and discs coated with CDP and SPC 89% and 90% respectively lower, than discs coated with the inert control ACP.

For calculations based on similarity law analysis, co-rotation of the water in the tank is an important consideration. Swirling and vortexing was greatly reduced in the open cubic tank compared with that in a cylindrical vessel, although moderate vortexing was still observed at higher velocities. The fixed construction of the rheometer does not enable the value of swirl factor to be directly determined. Roughness functions for the sandpaper surfaces could be collapsed onto a Nikuradse-type uniform sand curve by selecting a suitable value for the swirl factor once a suitable roughness scale had been fixed. The interdependence of these values limits the ability of this method to unambiguously provide roughness function data if the swirl factor cannot be experimentally determined, as there is a continuous range of pairings of values of this parameter and the surface roughness length factor that would give equally plausible fits. However, using the observed Nikuradse behaviour of well-characterised sandpaper surfaces to derive the swirl factor provides an acceptable estimate. Roughness functions for the fouled discs could be brought into alignment with Colebrook’s irregular surface roughness function by using the same value of swirl factor and assigning a suitable roughness height.

While the small scale of the minidisc rheometer system inevitably limits the scale of the fouling that can be accommodated, the data suggest it may be a useful method for assessing the rate of the initial steps of biomass accumulation and the potential for novel AF coatings to inhibit such fouling. The method is particularly suited for assessing biofilm fouling as the size and irregular placement of macrofouling organisms may introduce uncertainty in hydrodynamic analysis. Whether the calculated roughness functions for fouled surfaces could form a valid basis for ship scale drag predictions depends on establishing a relationship between the rotational flow in our tank and translational flow past a moving ship’s hull. This will be the subject of future studies.

## Supplementary Material

Supplemental Material

## Appendix

Table of Nomenclature and Units (Mass, Length, Time)

## Abbreviations

*Cm*: Moment or torque coefficient (-)
*M*: Torque (L2MT − 2)
*ρ*: Fluid density (ML − 3)
*ω*: Angular velocity = RPM × 2π/60 (T − 1)
*φ*: Swirl factor (0 < φ < 1) (-)
*ν*: fluid kinematic viscosity (L2T − 1)
*r*: Radius of the disc (L)
*d*: Diameter of the disc (2r) (L)
*b*: Thickness of the disc (L)
*Rer*: Rotational Reynolds number (-)
*ΔU+*: Roughness function (-)
*k+*: Roughness Reynolds Number (-)
$k_{s}^{+}$: Uniform sand roughness Reynolds Number (-)
*Ra*: 2D arithmetic surface roughness average (L)
*Rt*: 2D maximum peak to trough surface height (L)
*Sa*: 3D arithmetic surface roughness average (L)
*Sz*: 3D maximum peak to trough surface height (L)
