# Supplementary material for: A rapid benchtop method to assess biofilm on marine fouling control coatings
Source: Biofouling. 2021 Jun 21;37(4):452–64. doi: 10.1080/08927014.2021.1929937 (PMC8312500; doi:10.1080/08927014.2021.1929937)
Supplement: Supplemental Material [file GBIF_A_1929937_SM3454.pdf]

SUPPORTING INFORMATION

Data repository: Raw torque data for sandpapers and discs is available at (https://doi.org/10.5258/SOTON/D1589).

S1) COATING SURFACE ROUGHNESS

Roughness of unexposed coatings measured using Blue Light Interferometry

average um	Sa	SD (n=3)	Sz	SD (n=3)
ACP	5.27	0.05	76.3	1.5
FRC	6.07	0.25	107.8	14.6
CDP	5.57	0.10	92.3	15.0
SPC	4.17	0.05	130.4	3.9

Table S1.1 Surface roughness (Sa) of clean coatings

S2) SANDPAPER GRIT SIZES: According to FEPA (Fédération Européenne des Fabricants de Produits Abrasifs / Federation of European Producers of Abrasives). FEPA distinguishes between grain for sanding paper (FEPA P) and grain for sharpening stones or wheels (FEPA F). P Grits Conform to FEPA-Standards 43-1:2006, 43-2:2006. Particle size (µm) is determined by sieving Ref: https://www.fine-tools.com/G10019.html

FEPA P	Grit no.	um
**P40**	40	425
**P60**	60	269
**P120**	120	125
**P180**	180	82
**P240**	240	58.5

Table S2.1 Standard sandpaper roughness designations

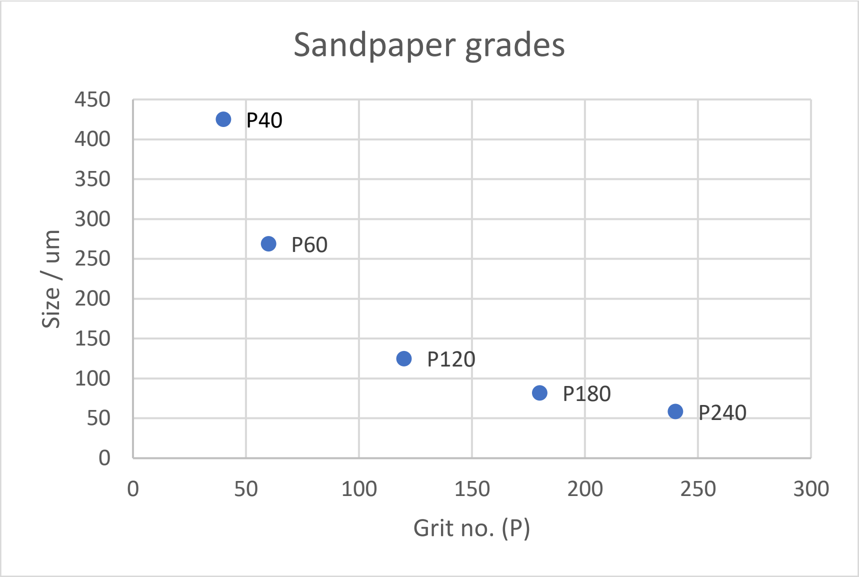

Fig. S2.2 Nominal abrasive grain size vs. grit number acc. to FEPA

S3) SANDPAPER SURFACE ROUGHNESS

	BLUE LIGHT Sa		ALICONA Sa		Overall	Alicona Sz	
Sandpaper	Mean	SD (n=10)	Mean	SD (n=10)	MEAN Sa	MEAN	SD (n=10)
grade	µm	µm	µm	µm	µm	µm	µm
P40	119	0.8	103	9.4	111	933	130
P60	70	0.5	65	6.8	68	511	104
P100	53	0.4	49	3.6	41	414	35
P120	35	0.1	28	3.5	32	268	22
P180	24	0.8	22	1.2	23	239	43
P240	15	0.2	17	2.0	16	174	18
Blank			8	0.9*		118	24

*n=5

Table S2.1 Sandpaper roughness measured by Blue Light and Alicona

S4) Wall shear stress of discs

Expressions for calculating wall shear stress from Granville (Granville 1973)

$$\tau_{\phi} = \frac{\rho\omega^2 r^2}{\left((4.96Log_{10}Re_r) - 5.74\right)^2}$$

$$\alpha = \frac{4.395}{(4.96Log_{10}Re_r) - 5.74} - 0.0107$$

$$\tau_r = \alpha\tau_\phi$$

$$\tau_w = \sqrt{{\tau_\phi}^2 + {\tau_r}^2}$$

ρ = 1024.5 kg·m^{-3}

r = 0.02 m

At maximum velocity

ω = 300 rad·s^{-1}

Rer = 7.0 x 10^4

Gives τ_w = 23.6 Pa

S5) ROUGHNESS HEIGHT EQUIVALENTS FOR FOULED DISCS.

Exposure time / days	30	85	110	176
ACP fouled / mm	0.001	0.035	0.420	0.600
FRC fouled / mm	0.002	0.003	0.130	0.150
CDP fouled / mm	0.002	0.002	0.037	0.004
SPC fouled /mm	0.001	0.001	0.030	0.001

Table S5.1 Roughness heights (Sz) for fouled discs needed to give agreement with Colebrook function.

S6) ONE-WAY ANOVA FOR COATINGS AFTER EACH EXPOSURE PERIOD

Software: Minitab 19 (Minitab LLC)

Factor Information

Factor	Levels	Values
Factor	4	ACP, FRC, CDP, SPC

Null hypothesis	All means are equal
Alternative hypothesis	Not all means are equal
Significance level	α = 0.05

Equal variances were not assumed for the analysis.

6 duplicates for each type of disc coating

Individual standard deviations (not pooled) are used to calculate the interval plots

S6.1 TORQUE VALUES FOR ANOVA

		M_1 / N.m					
30 days	ACP	7.78E-04	7.63E-04	7.82E-04	7.61E-04	7.76E-04	7.66E-04
	FRC	8.11E-04	7.96E-04	8.43E-04	7.68E-04	8.05E-04	8.06E-04
	CDP	8.12E-04	7.94E-04	8.20E-04	8.70E-04	7.90E-04	7.93E-04
	SPC	7.78E-04	7.86E-04	7.73E-04	7.70E-04	8.66E-04	7.87E-04
85 days	ACP	1.91E-03	1.65E-03	1.51E-03	1.33E-03	1.43E-03	1.27E-03
	FRC	9.11E-04	8.68E-04	8.86E-04	7.98E-04	8.30E-04	8.22E-04
	CDP	7.99E-04	7.87E-04	7.93E-04	8.26E-04	8.58E-04	7.88E-04
	SPC	7.82E-04	7.57E-04	7.40E-04	7.73E-04	7.62E-04	7.12E-04
110 days	ACP	4.95E-03	6.02E-03	7.01E-03	6.87E-03	6.34E-03	4.83E-03
	FRC	3.52E-03	3.38E-03	2.55E-03	1.72E-03	2.14E-03	1.72E-03
	CDP	1.65E-03	1.68E-03	1.87E-03	1.58E-03	1.20E-03	1.73E-03
	SPC	1.58E-03	1.56E-03	1.87E-03	1.53E-03	8.89E-04	2.34E-03
176 days	ACP	8.57E-03	9.18E-03	7.95E-03	1.06E-02	7.65E-03	3.83E-03
	FRC	1.39E-03	2.02E-03	1.75E-03	8.66E-04	2.26E-03	4.64E-03
	CDP	8.31E-04	8.43E-04	8.59E-04	8.95E-04	8.94E-04	1.06E-03
	SPC	7.39E-04	8.05E-04	7.52E-04	7.29E-04	7.50E-04	7.94E-04

Table S6.1 Torque of fouled disc faces (6 replicates for each coating) after fouling periods. Basis for ANOVA below.

S6.2 ANOVA - 30 Days Exposure

Welch's Test

Source	DF Num	DF Den	F-Value	P-Value
Factor	3	9.48039	5.96	0.015

Model Summary

R-sq	R-sq(adj)	R-sq(pred)
29.55%	18.98%	0.00%

Means

Factor	N	Mean	StDev	95% CI
ACP	6	0.000771	0.000009	(0.000762, 0.000780)

FRC	6	0.000805	0.000024	(0.000779, 0.000831)
CDP	6	0.000813	0.000030	(0.000782, 0.000845)
SPC	6	0.000793	0.000036	(0.000755, 0.000831)

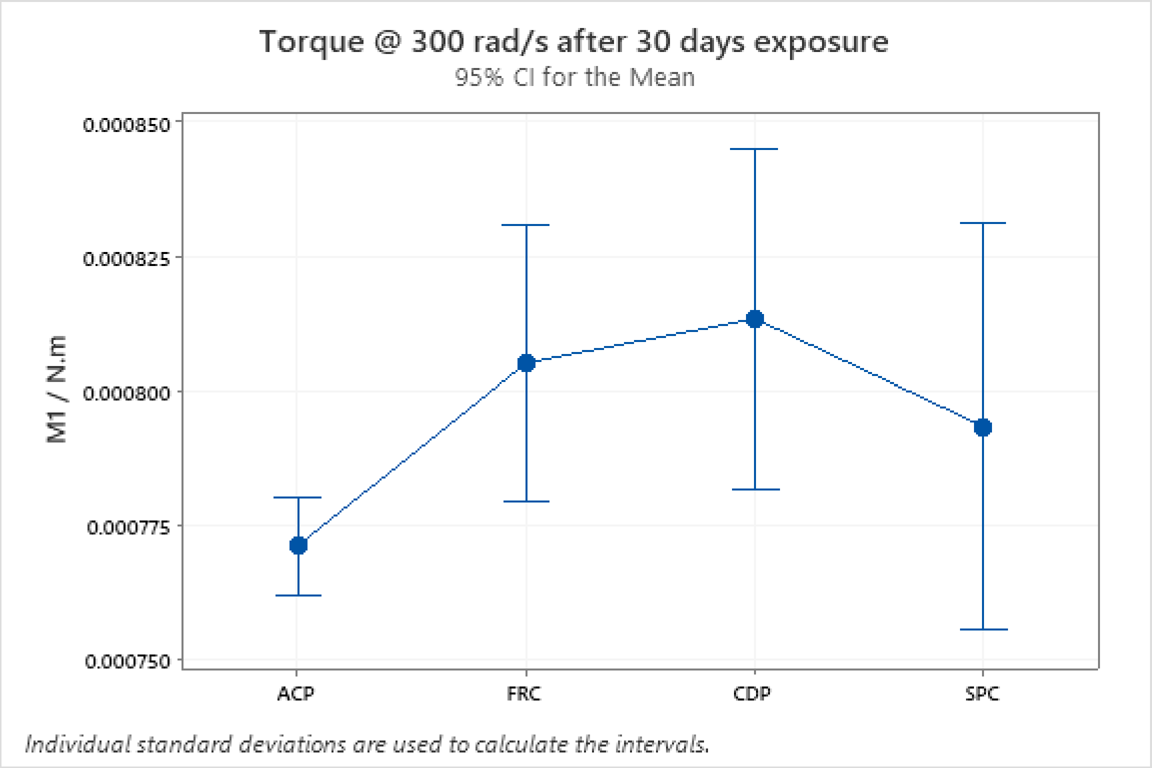

S6.3 ANOVA - 85 Days Exposure

Welch's Test

Source	DF Num	DF Den	F-Value	P-Value
Factor	3	10.5089	24.80	0.000

Model Summary

R-sq	R-sq(adj)	R-sq(pred)
88.66%	86.95%	83.66%

Means

Factor	N	Mean	StDev	95% CI
ACP	6	0.001514	0.000235	(0.001267, 0.001761)
FRC	6	0.000853	0.000043	(0.000807, 0.000898)
CDP	6	0.000808	0.000028	(0.000779, 0.000838)

SPC	6	0.000754	0.000025	(0.000728, 0.000781)

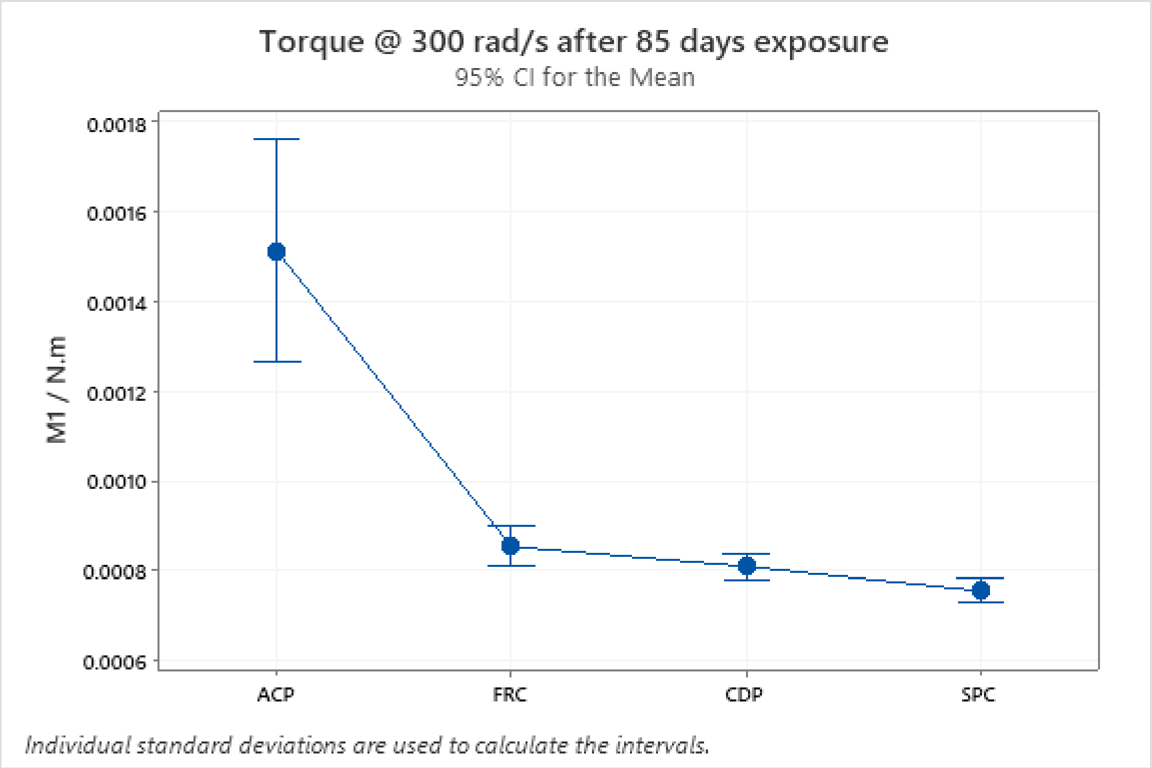

S6.4 ANOVA - 110 Days Exposure

Welch's Test

Source	DF Num	DF Den	F-Value	P-Value
Factor	3	9.73034	38.04	0.000

Model Summary

R-sq	R-sq(adj)	R-sq(pred)
89.76%	88.23%	85.26%

Means

Factor	N	Mean	StDev	95% CI
ACP	6	0.006003	0.000936	(0.005021, 0.006986)
FRC	6	0.002506	0.000795	(0.001673, 0.003340)
CDP	6	0.001619	0.000226	(0.001382, 0.001856)
SPC	6	0.001629	0.000475	(0.001130, 0.002127)

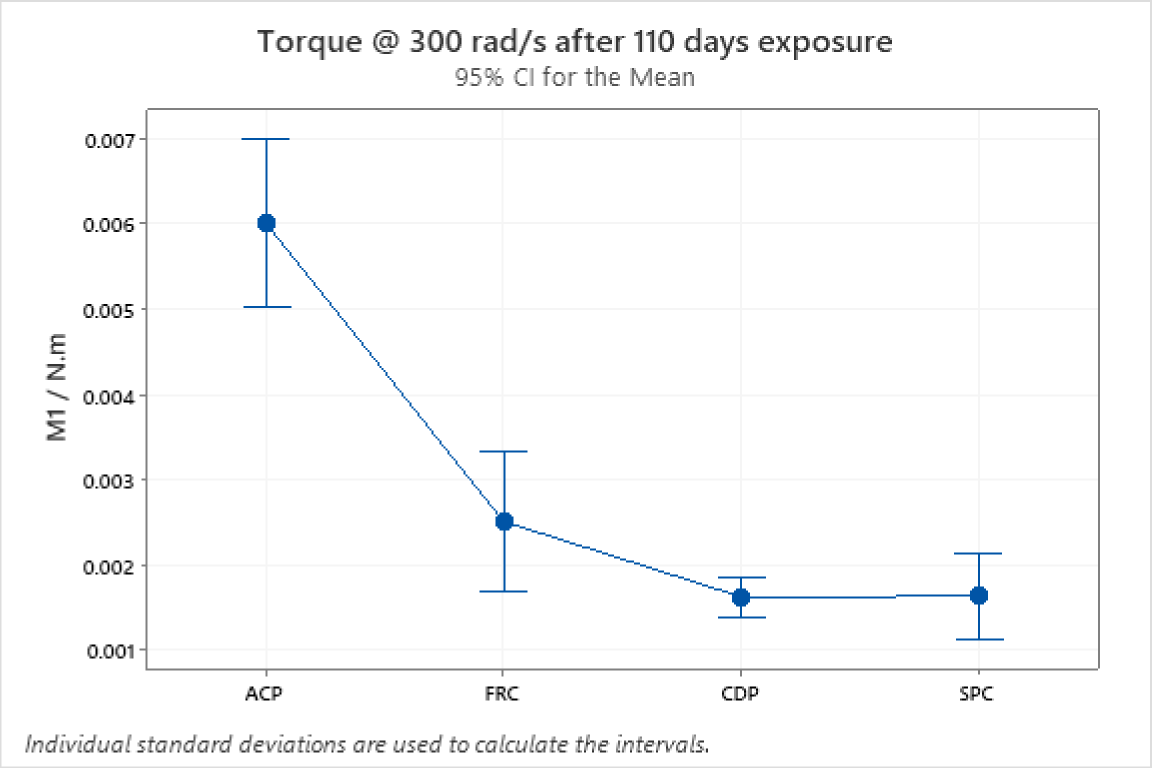

S6.5 ANOVA - 176 Days Exposure

Welch's Test

Source	DF Num	DF Den	F-Value	P-Value
Factor	3	8.96771	23.39	0.000

Model Summary

R-sq	R-sq(adj)	R-sq(pred)
85.81%	83.69%	79.57%

Means

Factor	N	Mean	StDev	95% CI
ACP	6	0.007958	0.002270	(0.005576, 0.010340)
FRC	6	0.002157	0.001313	(0.000779, 0.003535)
CDP	6	0.000897	0.000083	(0.000809, 0.000984)
SPC	6	0.000761	0.000031	(0.000729, 0.000794)

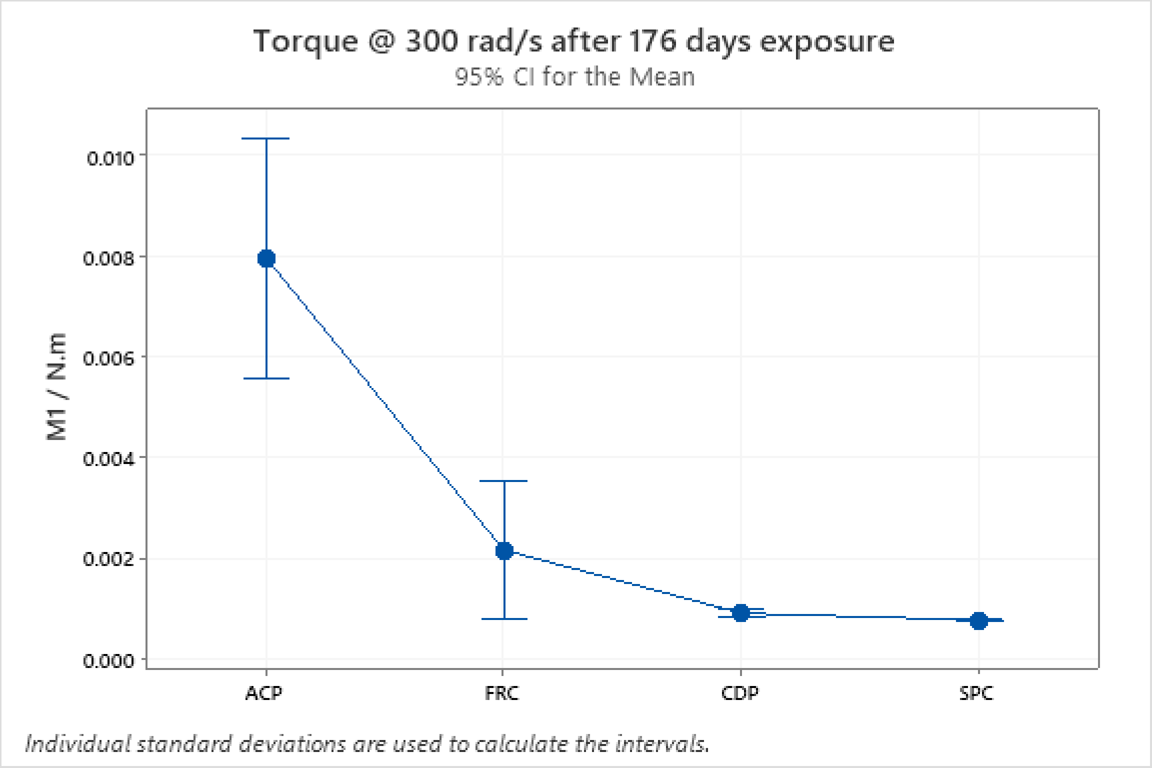
Torque @ 300 rad/s after 176 days exposure
95% CI for the Mean
M1 / N.m
0.010
0.008
0.006
0.004
0.002
0.000
ACP
FRC
CDP
SPC
Individual standard deviations are used to calculate the intervals.
